# Prevalence of Rheumatic Heart Disease Among School-Aged Children in Low- and Middle-Income Countries: A Systematic Review and Meta-Analysis

**DOI:** 10.7759/cureus.106833

**Published:** 2026-04-11

**Authors:** Miriam A Okorie, Collins C Okeke, Onyinye Ngige, Desmond E Orie, Otutochukwu O Ike-Obioha, Miliete T Berhe, Sylvahelen Okorienta, Euodia A Ugo-Ihanetu, Boluwatife E Olayemi, Oluwatosin A Shaba, Pace C Ejikem, Ephraim U Okeke

**Affiliations:** 1 Medicine and Surgery, Ebonyi State University, Abakiliki, NGA; 2 Internal Medicine, University of Port Harcourt Teaching Hospital, Port Harcourt, NGA; 3 Internal Medicine, Nnamdi Azikiwe University Teaching Hospital, Anambra, NGA; 4 Internal Medicine, Delta State University Teaching Hospital, Oghara, NGA; 5 Medicine and Surgery, University of Port Harcourt, Port Harcourt, NGA; 6 Medicine, Ayder Comprehensive Specialized Hospital, Mekelle University, Mekelle, ETH; 7 Public Health, Liberty University, Lynchburg, USA; 8 Internal Medicine, Rivers State University Teaching Hospital, Port Harcourt, NGA; 9 Internal Medicine, University of Medical Sciences Teaching Hospital, Ondo City, NGA; 10 Internal Medicine, Federal Medical Centre (FMC) Ebute Metta, Lagos, NGA; 11 General Practice, Afe Babalola University College of Medical and Health Science, Port Harcourt, NGA; 12 Psychiatry and Behavioral Sciences, Federal University Teaching Hospital (FUTH) Owerri, Owerri, NGA; 13 Internal Medicine, Enugu State University of Science and Technology Teaching Hospital, Enugu, NGA

**Keywords:** low- and middle-income country, prevalence, rheumatic heart diseases, school-aged children, school children

## Abstract

Rheumatic heart disease (RHD) is a preventable acquired valvular heart disease in children and young adults, which results from damage to the heart valves as a result of one or several episodes of rheumatic fever, resulting in progressive cardiac valvular damage and long-term morbidity. This systematic review and meta-analysis evaluate the pooled prevalence of RHD among school-age children in low- and middle-income countries (LMICs).

A comprehensive search was conducted from inception to the 5th of December, 2025, on PubMed and Google Scholar, and yielded 1,276 articles. Ten articles were included for analysis after undergoing screening using the predefined eligibility criteria. We included original research, published in a peer-reviewed journal, which talks about the prevalence of RHD in school-age children (six to 15 years) in any LMIC. We utilized a random-effects model, Cochrane Q statistic, I^2^ index, forest plot, and a funnel plot for meta-analysis. The Joanna Briggs Institute (JBI) critical appraisal tool was used for quality assessment of the included studies.

A total of 25,292 school-age children participated in this study across eight LMICs, with a mean age ranging from nine to 14 years. Among diagnosed cases, 130 were male, and 152 were female. The pooled prevalence of RHD was 2.46% (95% CI: 1.15%-4.25%), with sustainability heterogeneity observed across studies (I² = 98.3%, p < 0.001).

These findings indicate a continued burden of RHD among school-aged children in LMICs. The observed variability in prevalence suggests regional differences and potential gaps in early detection and primary preventive strategies. There is a need to strengthen screening and prophylactic programs in high-burden settings.

## Introduction and background

Rheumatic heart disease (RHD) is a preventable acquired valvular heart disease in children and young adults that develops following one or two episodes of acute rheumatic fever, an autoimmune inflammatory reaction to infection with streptococcal pharyngitis [1-3]. RHD is a sequela of an abnormal autoimmune response to group A streptococcus (GAS) that causes acute rheumatic fever, which occurs around three weeks after an untreated GAS pharyngitis infection. After multiple episodes of rheumatic fever, progressive fibrosis of heart valves can occur, which can lead to rheumatic valvular heart disease, and if left untreated, it may progress to heart failure and premature mortality [2,4,5]. Common symptoms of RHD include difficulty breathing, chest pain, swelling of the feet/ankle and a heart murmur [6]. The possible complications of RHD in children include permanent heart damage, heart failure, acute or chronic heart valve disease, and endocarditis. RHD imposes a substantial clinical and socioeconomic burden among school-aged children (six to 15 years) living in low- and middle-income countries (LMICs), contributing to poor school attendance, increased morbidity, and reduced school engagement. Limited access to early diagnosis, secondary prophylaxis, and specialized cardiac care in many low-resource settings contributes to persistent disease burden [7,8]. This systematic review and meta-analysis aimed to estimate the pooled prevalence of RHD among school-aged children in LMICs.

## Review

Methods

This systematic review was conducted in accordance with Preferred Reporting Items for Systematic Reviews and Meta-Analysis (PRISMA 2020). The study protocol was registered with PROSPERO (CRD420261296212). This study protocol was registered before data extraction.

Eligibility

Eligible studies were original peer-reviewed articles reporting the prevalence of RHD among school-aged children (six to 15 years) in countries classified as low or middle income by the World Bank.

Exclusion Criteria

Studies were excluded if they did not report prevalence data specific to school-aged children (six to 15 years). We also excluded studies involving patients >18 years of age, non-English publications, conference abstracts, reports, commentaries, case reports, case series, editorials, systematic reviews, and meta-analyses.

Search Strategy

A search was done from inception to the 5th of December 2025, on PubMed and Google Scholar (displayed only 50 pages) databases with the following search phrases across the database: (Prevalence) AND (rheumatic heart disease) AND (children) AND (low-income countries), "Prevalence" "rheumatic heart disease" "children" "low-income countries". More details are shown in Appendix 1.

The search results from various databases were imported into Rayyan referencing manager (Qatar Computing Research Institute, Doha, Qatar) [9], where duplicate, title, and abstract screening were carried out by three independent co-authors. Following abstract screening, the eligible articles were subjected to full-text screening using the pre-defined eligibility criteria. Disagreements were discussed among the authors, and another co-author will be invited if no resolution is reached.

Data extraction from the eligible articles was done by four co-authors independently into a Google spreadsheet. The extracted baseline variables include: author's name, country, study year, sample size, gender, mean age, children with RHD, and prevalence. The methodological quality of the included studies was assessed using the Joanna Briggs Institute (JBI) critical appraisal checklist for cross-sectional and cohort studies [10]. Nine cross-sectional studies and one cohort study were evaluated. The cross-sectional study demonstrated high methodological quality, all studies clearly defined the inclusion criteria, described study populations and settings in details and used a valid method of measuring exposure and outcomes. Potential cofounding factors were identified with appropriate strategies reported to address them. The cohort study also demonstrated generally good methodological quality; the exposed and unexposed groups were recruited from a similar population. The exposure and outcome measurements were conducted using a valid method. Some limitations were observed, including the lack of detailed reporting on follow-up duration, completeness of follow-up, and strategies to address loss to follow-up. The JBI critical appraisal tool does not have a standardized grading to report articles with high or low risk of bias. Despite these limitations, the overall methodological quality of the included studies was considered moderate to high, with a generally low risk of bias, as shown in Appendices 2 and 3.

The World Bank classification of LMICs is based on gross national income (GNI) per capita between $1,146 and $4,515.

Data Synthesis and Transformation

The primary outcome of interest was the pooled prevalence of RHD among school-aged children. Because prevalence rates (proportions) often approach the boundaries of 0 or 1, a Freeman-Tukey double arcsine transformation was applied to all raw proportions before pooling. This method stabilizes the variance and ensures that the resulting confidence intervals are restricted to the logical range of 0 to 100%, minimizing the bias introduced by studies with very small or very large estimates.

Meta-Analysis Model

Given the anticipated clinical and geographical diversity between the study settings (ranging across Africa, Asia, and Central America), we utilized a random-effects model (DerSimonian-Laird approach). This model assumes that the included studies represent a random sample from a larger population of possible studies and accounts for both within-study and between-study variance (τ2).

Assessment of Heterogeneity

Statistical heterogeneity was quantified using the Cochrane Q statistic and the I2 index. We interpreted I2 values of 25%, 50%, and 75% as representing low, moderate, and high heterogeneity, respectively. A p-value < 0.10 for the Q statistic was considered indicative of significant heterogeneity.

Publication Bias

Potential publication bias and small-study effects were assessed visually via a funnel plot of the transformed effect sizes against their standard errors. Statistical symmetry was further evaluated to identify if the pooled estimate was disproportionately influenced by small studies with high prevalence.

Subgroup Analysis and Moderators

To explore potential sources of heterogeneity, we performed a subgroup analysis based on geographical region (Africa vs. Non-Africa). Additionally, exploratory meta-regression was conducted using "Publication Year" and "Mean Age" as continuous covariates to determine their impact on the reported prevalence rates.

Results

**Figure 1:**
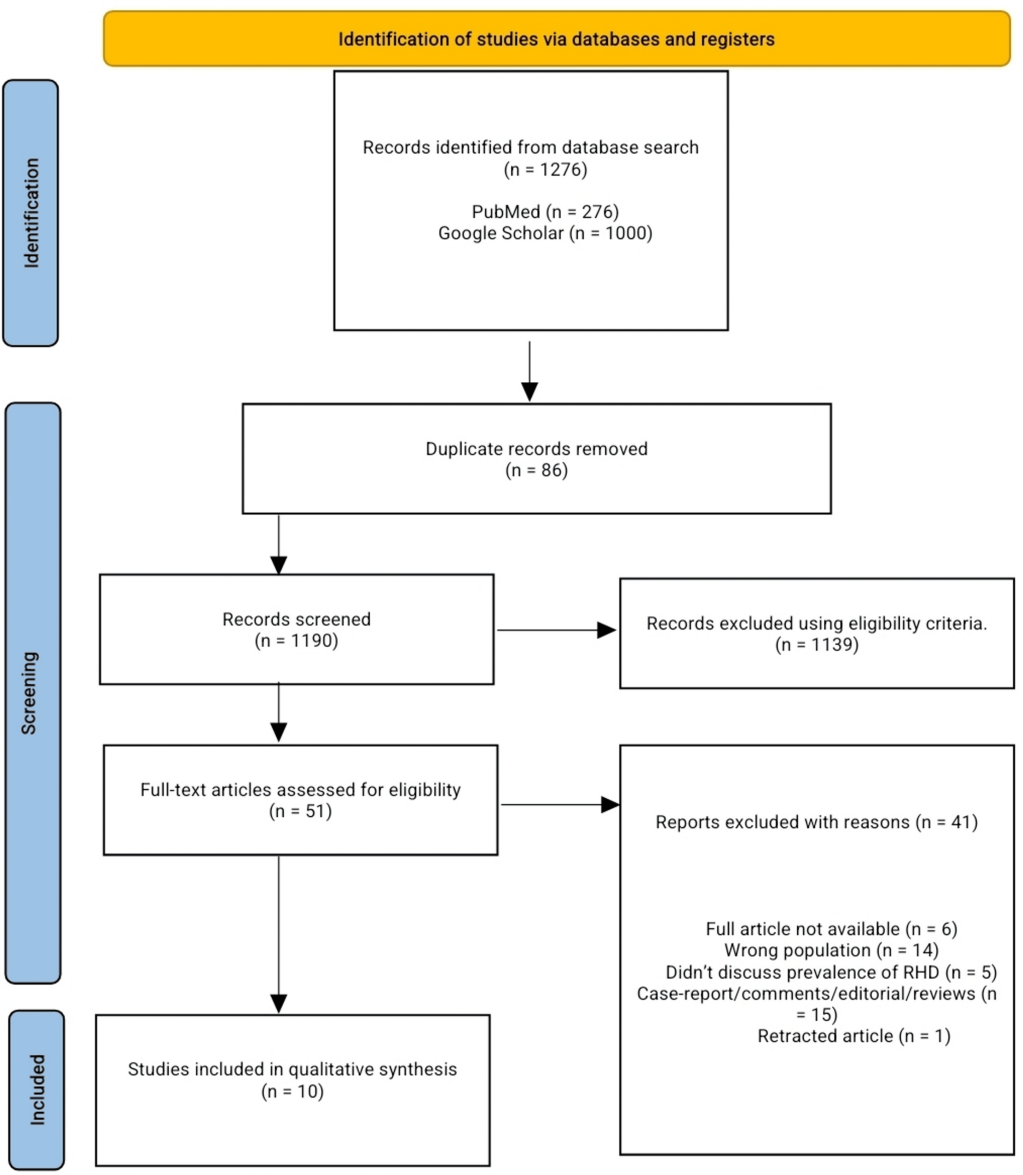
Preferred Reporting Items for Systematic Reviews and Meta-Analysis (PRISMA) flow-diagram RHD: Rheumatic heart disease

Our search across databases yielded 1,276 articles; 86 duplicates were removed, 1,190 articles were screened for title and abstract, and 1,139 articles were excluded following our predefined eligibility criteria. Fifty-one articles underwent full-text screening for possible inclusion in the final qualitative analysis and data extraction; 10 articles were included for analysis. We excluded six articles due to the unavailability of the full article, 14 articles with the wrong population (high-income countries and patients > 18 years), five articles that did not discuss RHD or prevalence, 15 articles were comments, reviews, editorials, case reports, and one retracted article. Full details of the PRISMA flow diagram are shown in Figure 1 below.

Study Characteristics

A total of 25,292 school-age children participated in this study across eight LMICs. Most of the included studies were conducted in African countries (Nigeria, Ethiopia, Uganda, Tanzania, and Egypt), with additional studies from Asia (Nepal and India) and Central America (Nicaragua). The mean age ranged from nine to 14 years, and among diagnosed cases, 130 were male, and 152 were female. Study duration ranges from 2003 to 2022. Echocardiography was the imaging diagnosis of choice across all studies. Full details of the study characteristics are shown in Table 1 below.

**Table 1:** Study Characteristics RHD: rheumatic heart disease, M: male, F: female.

Authors name	Country	Study year	Sample size	Mean age	Children with RHD	Prevalence per 1,000	Gender prevalence
Yadeta et al. [11]	Ethiopia	2016	3238	14	44	(14/1000)	M: 18 F: 26
Animasahun et al. [12]	Nigeria	2018	1846	9	36	(1.1per 10,000)	M:22 F:14
Gemechu et al. [13]	Ethiopia	2016	987	13	37	(37.5/1000)	N/A
Mapelli et al. [14]	Uganda	2022	163	9	14	(6.1/1000)	N/A
Kazahura et al. [15]	Tanzania	2021	949	10	32	(34/1000)	M:9 F:23
Nkereuwem et al. [16]	Nigeria	2020	417	14	9	(21.6/1000)	M:7 F:2
John A. Paar et al. [17]	Nicaragua	2010	3150	9	150	(48/1000)	M:67 F:83
Bahadur et al. [18]	Nepal	2003	9420	12	11	(1.2/1000)	M:7 F:4
Bigesh Nair et al. [19]	India	2015	2060	12	12	(5.83/1000)	N/A
Kotit et al. [20]	Egypt	2017	3062	10	95	(31/1000)	N/A

Meta-Analysis of Pooled Prevalence

A meta-analysis was conducted to estimate the pooled prevalence of RHD using a random-effects model with Freeman-Tukey double arcsine transformation. The overall pooled prevalence of RHD was 2.46% (95% CI: 1.15% to 4.25%). However, significant statistical heterogeneity was observed (I² = 98.3%, Cochrane’s Q = 515.23, df = 9, p < 0.001), indicating substantial variation in disease burden across different geographical settings.

**Figure 2:**
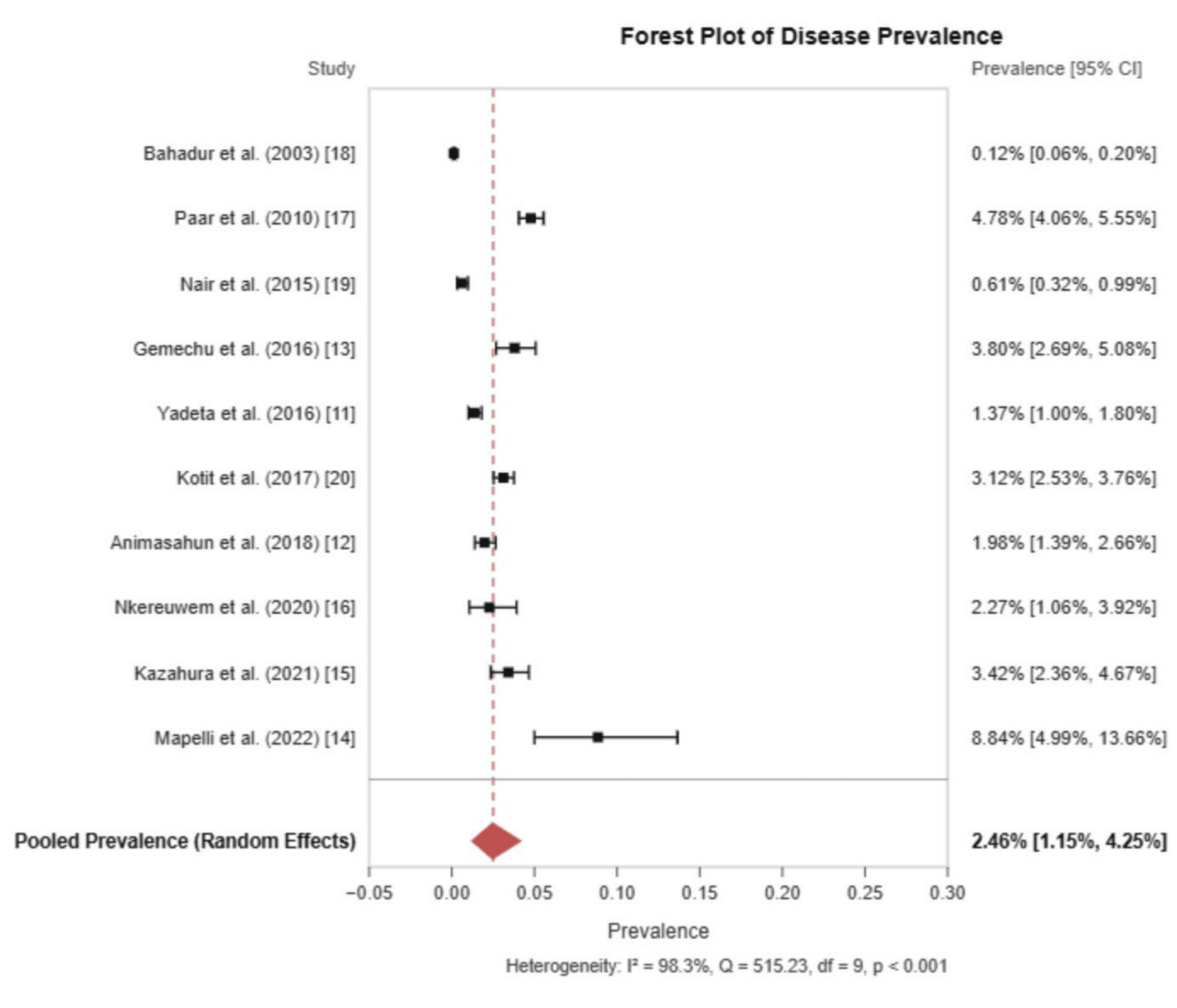
Forest plot of pooled prevalence of RHD RHD: Rheumatic heart disease.

The individual study results showed a wide range of prevalence estimates, from 0.12% (95% CI: 0.06%-0.21%) in Nepal (Bahadur et al.) to 8.59% (95% CI: 4.77%-13.97%) in Uganda (Mapelli et al.). This variability highlights the diverse disease burdens across regions and underscores the need for region-specific strategies to address RHD. The forest plot shown in Figure 2 below provides a clear visual summary of how RHD prevalence varies across studies and how these combine into an overall estimate for easier interpretation.

Assessment of Publication Bias 

**Figure 3:**
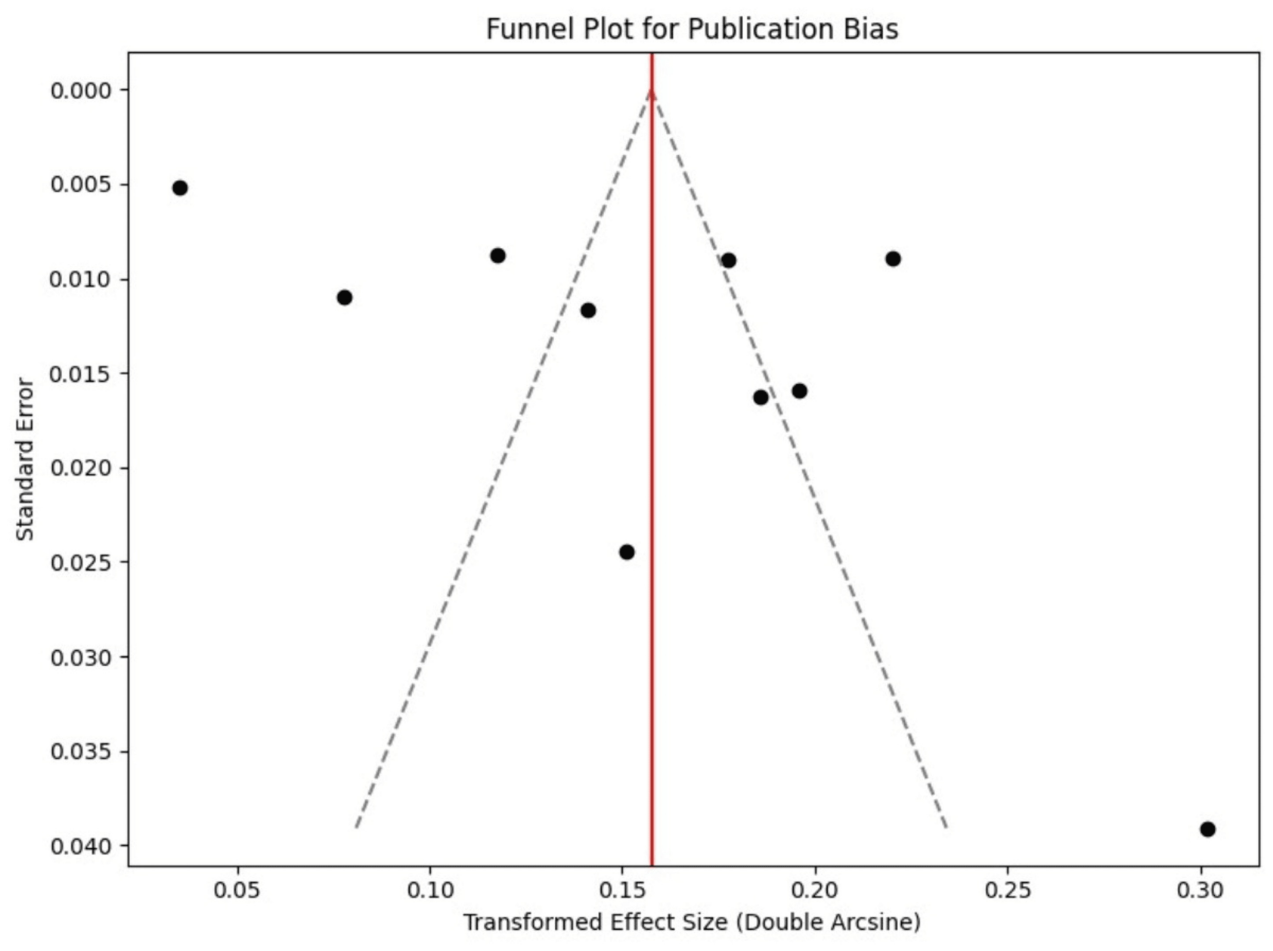
Funnel plot of the included studies.

The risk of publication bias was assessed via a funnel plot of the transformed effect sizes against their standard errors. Visual inspection of the funnel plot revealed asymmetry, with smaller studies appearing to report higher prevalence rates than larger studies. This suggests the potential presence of small-study effects or localized high-burden reporting, as shown in Figure 3 below in the funnel plot, which evaluates the reliability of our meta-analysis result and observes for overestimation due to bias in the available studies.

Subgroup Analysis by Geographical Region

A subgroup analysis was conducted to explore the sources of statistical heterogeneity, categorizing studies into African and non-African regions. The African region, comprising seven studies, showed a significantly higher pooled prevalence of RHD at 3.01% (95% CI: 1.63% to 4.81%), with high heterogeneity (I2 = 96.8%, p < 0.001) driven by variations between North African (Egypt: 3.10%) and East African (Uganda: 8.59%) estimates.

In contrast, the non-African region (three studies: India, Nepal, Nicaragua) had a lower pooled prevalence of 1.45% (95% CI: 0.00% to 5.25%), with extreme heterogeneity (I2 = 99.4%, p < 0.001) due to differences between low-prevalence Asian studies (Nepal: 0.12%, India: 0.58%) and Nicaragua's high-prevalence estimate (4.76%).

Comparing subgroups, the test for differences suggested that regionality alone doesn't fully explain the variance, indicating additional socio-economic or methodological factors at play. Table 2 below shows details of the subgroup analysis.

**Table 2:** Subgroup Analysis by Geographical Region

Subgroup	No. of Studies	Total Participants (N)	Pooled Prevalence (95% CI)	Heterogeneity (I2)
Africa	7	10,662	3.01% (1.63%–4.81%)	96.8%
Non-Africa	3	14,630	1.45% (0.00%–5.25%)	99.4%
Overall	10	25,292	2.46% (1.15%–4.25%)	98.3%

Meta-Regression (Exploratory)

An exploratory meta-regression was conducted to examine the influence of study year and mean age on RHD prevalence. The analysis found no significant association between publication year and prevalence (β=−0.002,p=0.64), indicating that the reported burden has remained relatively stable over the last two decades. Regarding mean age, a marginal trend suggested higher prevalence in older cohorts, but this didn't reach statistical significance (p=0.12).

Discussion

This systematic review and meta-analysis provide updated evidence on the prevalence of RHD among school-aged children in LMICs, revealing a pooled prevalence of 2.46% (95% CI: 1.15-4.25%). This finding underscores that RHD remains a substantial public health challenge in resource-limited settings, despite global efforts toward elimination. The high degree of heterogeneity (I² = 98.3%) observed across studies reflects wide disparities in socioeconomic contexts, diagnostic criteria, and access to preventive care across regions.

Regional and Epidemiologic Variation

Marked variation in RHD prevalence, ranging from 0.12% in Nepal [18] to 8.59% in Uganda [14], highlights the disease’s geographical concentration in sub-Saharan Africa and parts of South Asia. This pattern is consistent with recent global estimates, which report the highest RHD prevalence in East Africa (8.2 per 1,000) and persistently high rates in South Asia (5.1 per 1,000) [21,22]. The higher prevalence observed in several African settings may be associated with limited access to primary healthcare, delayed diagnosis, and suboptimal delivery of penicillin prophylaxis [23,24]. Conversely, lower rates reported in Asian studies may reflect improvements in school-based screening, antibiotic stewardship, and early GAS infection control programs [25].

Heterogeneity and Diagnostic Advances

The substantial heterogeneity in pooled estimates arises from methodological diversity and evolving diagnostic standards. All included studies employed echocardiographic screening, the current gold standard, but differences in image quality, operator training, and guidelines applied may have influenced case detection [26,27]. The Freeman-Tukey double arcsine transformation employed in this meta-analysis appropriately stabilized variance among studies with small sample sizes or low prevalence, yet genuine epidemiologic variability persists, thus reflecting real differences in community disease burden.

Technological advances in portable and handheld echocardiography have revolutionized screening capacity in low-resource settings, facilitating detection of subclinical RHD [28]. However, as recent multicountry initiatives have shown, standardization of echocardiographic criteria and operator certification is crucial to ensure diagnostic reliability and comparability across regions [29].

Public Health Implications

The pooled prevalence of 2.46% suggests that in high-burden settings, up to one in 40 school-aged children in LMICs are affected by RHD. This figure exceeds estimates from the Global Burden of Disease analysis, which reported a lower global prevalence and highlighted persistent inequities in RHD distribution [30,31]. These disparities emphasize the socioeconomic determinants of RHD, including poverty, overcrowding, and limited access to antibiotics, therefore mirroring findings from Watkins et al. [21] and Abram et al. [32].

A slight female predominance (152 females vs 130 males) was observed across studies, which is consistent with prior evidence of gender disparities in healthcare access, delayed care-seeking behavior, and higher rates of progression from subclinical to clinical disease in girls [30,31]. Addressing these disparities requires gender-sensitive school health policies and equitable access to preventive care.

Publication Bias and Data Limitations

Visual inspection of the funnel plot demonstrated asymmetry, with smaller studies reporting higher prevalence estimates. This may reflect small-study effects or preferential publication from regions with recognized high RHD burden, as similarly noted by Watkins et al. [21] and Ghamari [22]. The exclusion of non-English-language publications and limited representation from Central Africa and Southeast Asia remain key limitations that warrant future multicountry, prospective surveillance studies.

Moreover, several included studies had a moderate-to-high risk of bias due to non-random sampling or reliance on single screening rounds. Standardized multicentric designs with longitudinal follow-up are necessary to clarify true incidence and progression dynamics.

Policy and Research Implications

Achieving the WHO [23] and World Heart Federation [33] RHD elimination target will require integrated strategies emphasizing both primary prevention (GAS pharyngitis control) and secondary prevention (benzathine penicillin prophylaxis). Embedding RHD programs within broader non-communicable disease (NCD) frameworks, as proposed by Leon et al. [34], could improve sustainability. Emerging technologies such as AI-assisted diagnosis have been proposed in echocardiographic interpretation, telemedicine, and mobile health screening, offering opportunities to enhance diagnostic coverage and follow-up, particularly in remote schools [35,36].

Strengths and Limitations

This study is one of the few recent systematic reviews and meta-analyses to comprehensively estimate the pooled prevalence of RHD among school-aged children in LMICs using echocardiographic confirmation as the diagnostic standard. A major strength lies in the structured search strategy that spanned multiple databases and adhered to PRISMA 2020 guidelines, ensuring comprehensive coverage and methodological transparency. The use of a random-effects model with Freeman-Tukey double arcsine transformation allowed for stable variance estimates in the presence of substantial heterogeneity. Furthermore, the inclusion of only echocardiography-based studies enhanced diagnostic accuracy and comparability, while stratified analysis across diverse low-income regions provided a realistic reflection of global disparities in RHD burden.

However, several limitations must be acknowledged. First, significant statistical heterogeneity (I² = 98.3%) indicates considerable variation in study populations, diagnostic criteria, and screening intensities, which may limit generalizability. Second, publication bias was evident from funnel plot asymmetry, suggesting that smaller studies with higher prevalence may be overrepresented. Third, the exclusion of non-English studies and those without accessible full texts may have led to underrepresentation of data from some high-burden settings. In addition, variations in sample size, screening methodologies, and definitions of borderline RHD introduce potential misclassification bias. Lastly, the cross-sectional nature of most included studies limits inference on incidence, progression, or the effectiveness of preventive interventions. Despite these constraints, the study offers a valuable synthesis of contemporary evidence and highlights critical gaps to inform future research and global elimination strategies.

Recommendations

The findings of this meta-analysis underscore the urgent need for a coordinated global response to reduce the burden of RHD among school-aged children in LMICs. National health authorities should align their strategies with the WHO Operational Framework for the Elimination of RHD (2023-2030) and the World Heart Federation’s Roadmap, integrating RHD prevention into existing NCD and school health programs. These findings support consideration of targeted school-based screening programs in high-burden settings.

Ensuring the consistent availability of benzathine penicillin G (BPG) remains central to both primary and secondary prevention. Governments should strengthen procurement and supply chains and empower community health workers to deliver sore throat management and follow-up care at the primary level. Health systems should also integrate RHD management into NCD and cardiac care platforms, using task-shifting to nurses and mid-level providers in resource-limited settings.

The adoption of digital innovations, such as tele-echocardiography and AI-assisted diagnostics, can improve screening coverage and diagnostic accuracy in remote areas. Gender-responsive strategies should ensure equal access to prevention and treatment, particularly for adolescent girls who face higher disease risk. Finally, multicountry RHD surveillance networks and cost-effectiveness research are needed to monitor progress, refine interventions, and sustain elimination efforts.

Achieving the 2030 RHD elimination target will require political commitment, sustained investment, and regional collaboration to deliver equitable, evidence-based prevention and care for all children at risk.

## Conclusions

This systematic review and meta-analysis demonstrate that RHD remains prevalent among school-aged children in LMICs, with substantial variability across regions. Pooled prevalence indicates a continued burden in several African and Asian countries. Strengthening early detection and preventive strategies in high-risk regions may help reduce long-term morbidity and support global elimination efforts.

## Appendices

Appendix 1 

**Table 3:** Search strategy

Database	Search Query	Outcome
PubMed	(((Prevalence) AND (rheumatic heart disease)) AND (children)) AND (low-income countries)	276
Google scholar	"Prevalence" "rheumatic heart disease" "children" "low-income countries"	1000

Appendix 2 

**Table 4:** Joanna Briggs Institute (JBI) critical appraisal tool for cross-sectional studies

Checklist question	Yadeta et al [11].	Gemechu et al [13].	Mapelli et al [14].	Kazahura et al [15].	Nkereuwem et al [16].	John A. Paar et al [17].	Bahadur et al [18].	Bigesh Nair et al [19].	Kotit et al [20].
Were the criteria for inclusion in the sample clearly defined?	Y	Y	Y	Y	Y	Y	Y	Y	Y
Were the study subjects and the setting described in detail?	Y	Y	Y	Y	Y	Y	Y	Y	Y
Was the exposure measured in a valid and reliable way?	Y	Y	Y	Y	Y	Y	Y	Y	Y
Were objective, standard criteria used for measurement of the condition?	Y	Y	Y	Y	Y	Y	Y	Y	Y
Were confounding factors identified?	Y	Y	Y	Y	Y	Y	Y	Y	Y
Were strategies to deal with confounding factors stated?	Y	Y	Y	Y	Y	Y	Y	Y	Y
Were the outcomes measured in a valid and reliable way?	Y	Y	Y	Y	Y	Y	Y	Y	Y
Was appropriate statistical analysis used?	Y	Y	Y	Y	Y	Y	Y	Y	Y

Appendix 3 

**Table 5:** Joanna Briggs Institute (JBI) critical appraisal tool for cohort study

Checklist question	Animasahun et al [12].
Were the two groups similar and recruited from the same population?	Y
Were the exposures measured similarly to assign people to both exposed and unexposed groups?	Y
Was the exposure measured in a valid and reliable way?	Y
Were confounding factors identified?	Y
Were strategies to deal with confounding factors stated?	Y
Were the groups/participants free of the outcome at the start of the study (or at the moment of exposure)?	Y
Were the outcomes measured in a valid and reliable way?	Y
Was the follow up time reported and sufficient to be long enough for outcomes to occur?	N
Was follow up complete, and if not, were the reasons to loss to follow up described and explored?	N
Were strategies to address incomplete follow up utilized?	N
Was appropriate statistical analysis used?	Y
